# Long-term integrative personalized medicine care in massive brainstem hemorrhage: A CARE-compliant case report

**DOI:** 10.1097/MD.0000000000050954

**Published:** 2026-10-02

**Authors:** Ye-Vin Lee, Jonghun Ahn, Euiju Lee, Chelin Park, Eui-Jin Son, Ja Yeon Jeong

**Affiliations:** a College of Korean Medicine, Kyung Hee University, Seoul, Republic of Korea; b Department of Sasang Constitutional Medicine, Kyung Hee University College of Korean Medicine, Kyung Hee University Korean Medicine Hospital, Seoul; c Department of Clinical Korean Medicine, Graduate School, Kyung Hee University, Seoul, Republic of Korea.

**Keywords:** inflammation, integrative personalized medicine care, long-term herbal medicine, massive brainstem hemorrhage, safety

## Abstract

**Rationale:**

Brainstem hemorrhage is a rare but devastating subtype of intracerebral hemorrhage that frequently presents with impaired consciousness, quadriplegia, and recurrent infections. Integrative personalized medicine care (IPMC), which combines essential conventional treatments with personalized traditional Korean medicine interventions, has been suggested to improve functional outcomes and support immune regulation. By closely monitoring a patient with massive brainstem hemorrhage treated with IPMC, we observed neurological improvement, effective control of recurrent infections and inflammation, and sustained safety of long-term herbal medications.

**Patient concerns:**

A 48-year-old female patient with a brainstem hemorrhage was managed at Kyung Hee University Hospital with IPMC for posthemorrhagic functional impairment and recurrent infection.

**Diagnoses:**

The patient was diagnosed with a massive pontine hemorrhage with posthemorrhagic functional impairment and recurrent infection.

**Interventions:**

The patient received conventional treatment and integrative personalized traditional Korean medicine interventions, including herbal medications and acupuncture.

**Outcomes:**

We retrospectively analyzed the 2-year clinical course of the patient who received IPMC to evaluate the efficacy and safety of the treatment. Functional outcomes and inflammation control were assessed using standardized scales (modified Barthel index, Korean mini-mental state examination, Glasgow Coma Scale, global deterioration scale, level of consciousness, manual muscle testing, and Fugl-Meyer assessment) and hematological markers (C-reactive protein, neutrophil-to-lymphocyte ratio, and systemic inflammation response index). Liver and renal functions were monitored through laboratory tests (alanine aminotransferase, aspartate aminotransferase, γ-glutamyltransferase, blood urea nitrogen, serum creatinine, and estimated glomerular filtration rate). Regarding the efficacy of IPMC, functional improvements in neurological and motor outcomes were observed throughout the treatment period. Dependence on antibiotics progressively decreased, whereas the proportion of herbal medication-centered treatments increased. Infections with C-reactive protein levels <2.0 mg/dL were effectively managed with herbal medications alone. Furthermore, despite the long-term use of combined treatments, no hepatotoxicity or nephrotoxicity was observed, confirming the safety of IPMC.

**Lessons:**

This case demonstrates the efficacy and safety of IPMC, which integrates herbal medications with conventional treatment, for managing posthemorrhagic functional impairment and recurrent infections. These findings provide a foundation for future prospective studies. Further research is required to strengthen this evidence and validate the effectiveness of IPMC.

## 1. Introduction

Brainstem hemorrhage is a rare but devastating type of intracerebral hemorrhage, accounting for 5% to 10% of cases and associated with high morbidity and mortality.^[1]^ The prognosis is closely related to the hematoma’s location and size, which is classified by Chung and Park into 4 types based on computed tomography (CT) findings: massive, bilateral tegmental, basal-tegmental, and small unilateral tegmental.^[2]^ Among these, the massive type is particularly fatal, with a reported survival rate of only 7.1%.^[2]^ Owing to the dense concentration of nuclei and ascending–descending tracts, patients frequently present with impaired consciousness, dysautonomia, respiratory failure, dysphagia, gaze palsy, and quadriplegia.^[3]^ In later phases, this type is highly prone to complications such as pneumonia, urinary tract infection (UTI), pressure injuries, and joint contractures: all of which prolong hospitalization and worsen functional outcomes.^[3]^ In this context, inflammation remains a critical driver of post-stroke complications, and biomarkers such as C-reactive protein (CRP), neutrophil-to-lymphocyte ratio (NLR), and systemic inflammation response index (SIRI) have emerged as valuable indicators in infections and cerebrovascular diseases.^[4]^

Alongside advances in neurocritical care, complementary approaches including herbal medications and acupuncture have been explored to improve recovery after hemorrhagic stroke, with studies reporting benefits in motor function, dysphagia, spasticity, and immune regulation.^[5–7]^ Although direct evidence for brainstem hemorrhage is limited, related intracerebral hemorrhage research suggests a potential role of integrative strategies.^[8]^ For instance, a 41-day inpatient case (from April 5, 2021, to May 15, 2021) of acute primary pontine hemorrhage with one-and-a-half syndrome was managed with integrative Korean–Western collaborative care, incorporating Korean medicine (herbal medications, acupuncture, and moxibustion), Western pharmacotherapy, and rehabilitation.^[9]^

Against this background, the present case is distinctive in applying integrative personalized medicine care (IPMC): a therapeutic strategy designed to address the complex challenges of brainstem hemorrhage with recurrent infections^[10]^: with a focus on evaluating functional recovery, inflammation control, and the long-term safety of herbal medications.^[11,12]^ Our team administered IPMC, consisting of conventional treatment combined with personalized herbal medications and acupuncture. By closely monitoring the patient’s condition in a hospital setting and managing potential interactions between herbal and conventional medications, we observed neurological improvement, effective control of recurrent infections and inflammation, and sustained safety with long-term herbal medications. This case report aimed to present these findings and discuss the mechanisms supporting the safety and efficacy of long-term integrative personalized medicine in patients with massive brainstem hemorrhage.

## 2. Case presentation

### 2.1. Patient information

The patient was a 48-year-old female, with a height of 160 cm and a weight of 55 kg, who had no significant past medical history. On September 21, 2022, she presented to the emergency department at Kyung Hee University Medicine Hospital with a sudden onset of headache followed by a rapid change in mental status. A brain CT (shown in Fig. 1A) performed 5 hours post-onset revealed an acute brainstem hemorrhage with significant perilesional edema and intraventricular hemorrhage occupying the third and fourth ventricles. The hematoma volume was approximately 5.6 mL, measured using the ABC/2 method^[13]^ (A = 32.13 mm, B = 20.35 mm, C = 17.09 mm). Following diagnosis, she was transferred to the hospital’s intensive care unit and remained admitted in the neurosurgery ward for 6 weeks. Thereafter, she was transferred to the Department of Sasang Constitutional Medicine at Kyung Hee University Korean Medicine Hospital for management with integrative personalized medicine, where she remained from November 23, 2022, to June 30, 2025.

**Figure 1.**
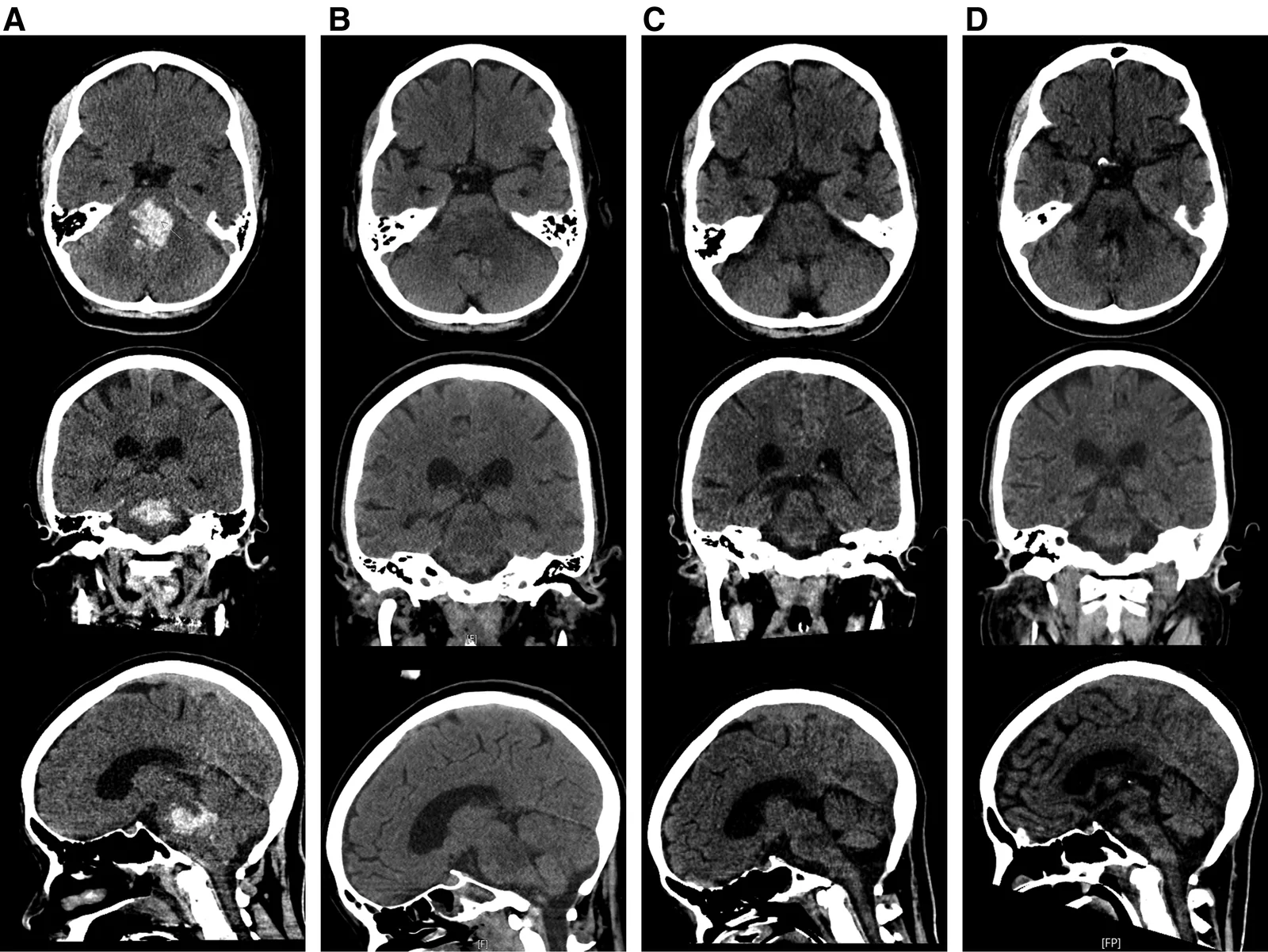
Sequential brain computed tomography (CT) scans. (A) The initial brain CT, performed 5 hours after symptom onset, identified an acute hemorrhage in the brainstem. Associated findings included marked perilesional edema and intraventricular hemorrhage (IVH) occupying the third ventricle, extending through the foramen of Magendie and filling the fourth ventricle. Follow-up CT scans obtained at (B) 1 month, (C) 2 months, and (D) 4 months demonstrate gradual resolution of the hematoma.

At the time of admission, the patient was in a drowsy state and presented with quadriplegia, characterized by an inability to move her upper extremities and only intermittent, slight movements in her lower extremities without the ability to lift them, accompanied by foot drop. Comorbidities included hypertension, diagnosed in 2022 during the ICU stay, and dyslipidemia, which was newly diagnosed during hospital stay in 2023. During hospitalization, she experienced ten infectious events, including UTI, pneumonia, and COVID-19. However, the family or social history was unremarkable.

Between June 21 and June 28, 2023, the patient was briefly transferred to the Department of General Surgery, for percutaneous endoscopic gastrostomy tube insertion. Excluding this period, she received IPMC, including herbal medications, acupuncture, rehabilitation, and conventional treatments, until discharge on June 30, 2025. The patient’s key medical problems and corresponding treatments are summarized in the timeline presented in Fig. 2.

**Figure 2.**
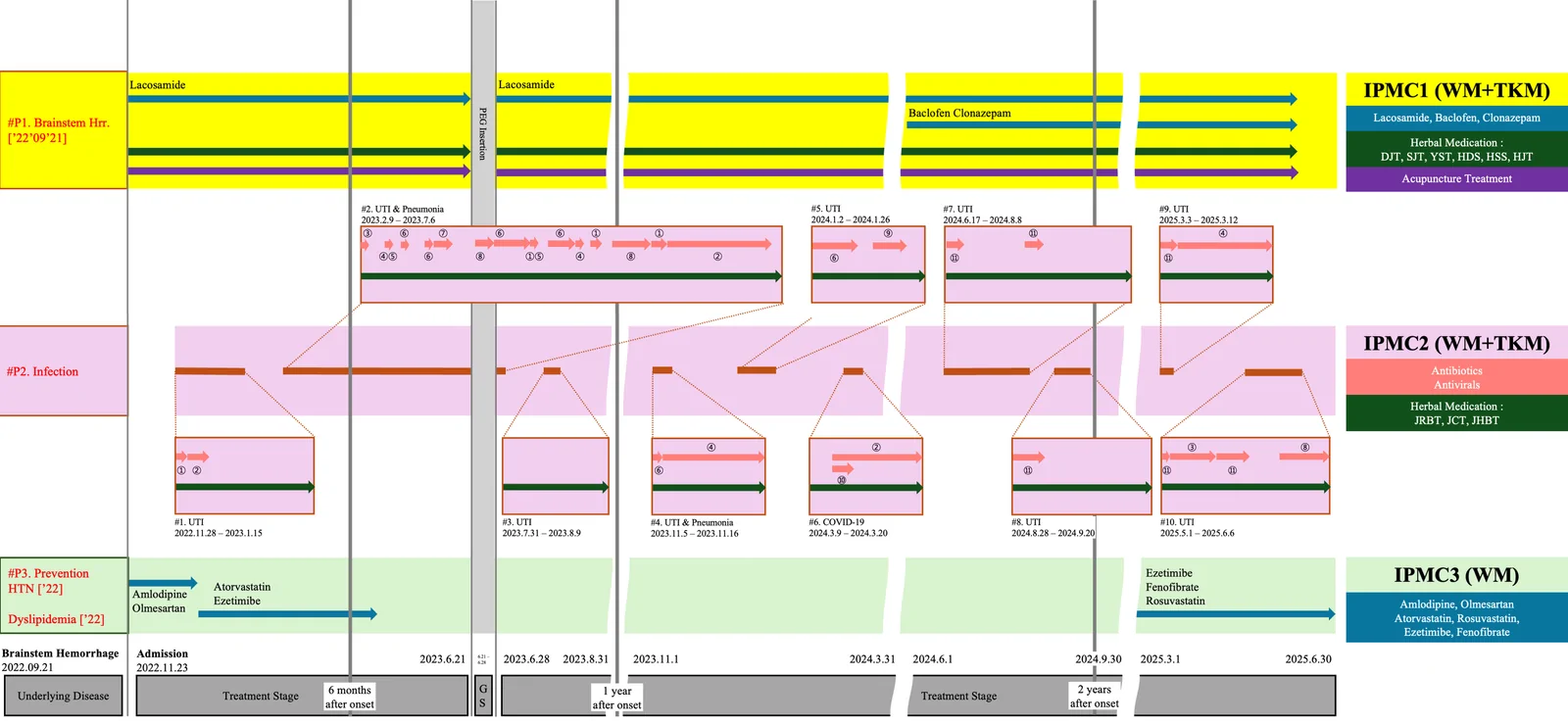
Clinical course of the patient’s management. The following arrows indicate treatment periods: green for herbal medicine; purple for acupuncture; blue for non-antimicrobial drugs; and pink for antimicrobials (antibiotics and antivirals). Numbered circles denote the specific antimicrobial agents administered: fluconazole, ceftriaxone, cefepime, meropenem, vancomycin, ertapenem, moxifloxacin, levofloxacin, teicoplanin, remdesivir, and ceftazidime. DJT = Dokhwaljihwang-tang, GS = gastrointestinal surgery, HDS = Hyeongbangdojeok-san, HJT = Hyeongbangjihwang-tang, HSS = Hyeongbangsabaek-san, IPMC = integrative personalized medicine care, JCT = Jeoryeongchajeonja-tang, JHBT = Jihwangbaekho-tang, JRBT = Jyeoryeongbaekho-tang, PEG = percutaneous endoscopic gastrostomy, SJT = Sipimijihwang-tang, TKM = traditional Korean medicine, UTI = urinary tract infection, WM = Western medicine, YST = Yanggyeoksanhwa-tang.

This study was conducted as a retrospective observational analysis. Owing to the nature of the research, which involved reviewing medical, prescription, and progress notes, obtaining prior informed consent from the patient was deemed impracticable. Moreover, considering the minimal risk posed to the patient and the absence of reasonable grounds to object to participation, the requirement for informed consent was waived. The study protocol was reviewed and approved by the Institutional Review Board (IRB) of Kyung Hee University Korean Medicine Hospital (IRB No. KOMCIRB 2025-09-010; Date of Approval: 2025-10-28).

### 2.2. Diagnostic assessment

Follow-up CT scans performed at 1 month (shown in Fig. 1B), 2 months (shown in Fig. 1C), and 4 months (shown in Fig. 1D) after symptom onset confirmed gradual resolution of the hematoma.

A complete blood count test conducted on November 23, 2022, revealed a white blood cell (WBC) count of 7.96 × 10^3^/μL, NLR of 0.24, and SIRI of 1.39. Serum chemistry tests conducted on November 28, 2022, revealed a CRP level of 0.99 mg/dL, aspartate aminotransferase (AST) level of 23 U/L, alanine aminotransferase (ALT) level of 17 U/L, γ-glutamyltransferase (GGT) level of 47 U/L, blood urea nitrogen (BUN) level of 18 mg/dL, serum creatinine (SCr) level of 0.39 mg/dL, and modification of diet in renal disease-estimated glomerular filtration rate of 176 mL/min/1.73 m^2^.

At the time of admission, quadriplegia and severe cognitive impairment were noted due to brainstem hemorrhage. Physical examinations were performed throughout hospitalization to assess patient condition. The manual muscle testing (MMT) score was Grade 1 on the right and Grade 2 on the left side, as assessed on November 23, 2022. The Korean mini-mental state examination score was 0, as assessed on January 12, 2023. Although a modified Barthel index (MBI) score was not recorded at the time of admission, progress notes from Nov 23, 2022, indicated an MBI score of 0, denoting total dependence. Similarly, the Fugl-Meyer assessment (FMA) score was 8 on both sides, as evaluated using the same progress notes, which confirmed severe motor impairment. The Glasgow coma scale (GCS) score was 10, and level of consciousness (LOC) was Grade II (drowsy) according to the progress notes dated November 23, 2022.

Based on these findings, the patient’s new primary pontine hemorrhage score was 3 (2 points for initial GCS 3 and 1 point for hematoma volume), indicating a 30-day mortality rate of 81.8%. Furthermore, the presence of massive bilateral hemorrhage, intraventricular extension, and the need for late tracheostomy (>7 days) indicated a markedly unfavorable prognosis.^[14]^

### 2.3. Diagnostic methods

To assess the efficacy of IPMC administered for over 2 years in the patients with brainstem hemorrhage, assessments were broadly divided into 2 main domains: functional outcomes and inflammation management related to complications. Functional outcomes were subdivided into neurological and motor outcomes. Neurological outcomes were assessed using the MBI, K-MMSE, global deterioration scale (GDS), GCS, and LOC. MBI scores were recorded 10 times at the Department of Rehabilitation Medicine from February 2, 2024, to July 1, 2025. K-MMSE scores were assessed 11 times from January 12, 2023, to July 1, 2025, and GDS scores were measured 7 times from February 8, 2023, to May 30, 2025. GCS scores and LOC grades were not evaluated during hospitalization; therefore, they were inferred from mental status descriptions in the progress notes.

Motor outcomes were evaluated using the MMT and FMA. MMT scores were assessed 11 times at the Department of Rehabilitation Medicine from January 11, 2023, to May 21, 2025, and also recorded daily in progress notes. Differences were noted between MMT scores recorded in the department and those in the progress notes. However, because the progress notes provided more detailed descriptions of the patient’s condition, MMT scores recorded in the progress notes were prioritized for analysis. Upper and lower limb MMT scores were based on the MMT 5-point scale, and joint-specific MMT scores were based on the modified MMT scale. For graphical representation, modified MMT scale scores of 2+, 2, and 2 were recorded as 2.5, 2 and 1.5, respectively. FMA scores were assessed twice at the Department of Rehabilitation Medicine on March 5, 2024, and May 28, 2025, as well as recorded in the progress notes at the time of admission to compare the conditions at admission and during hospitalization.

Infectious periods were defined based on combined laboratory and clinical criteria. The onset of infection was determined as the first day when CRP exceeded 1.0 mg/dL on at least 2 consecutive measurements, accompanied by a WBC count > 10 × 10^3^/μL and relevant clinical symptoms including fever, dysuria, or changes in sputum characteristics. Resolution of infection was defined as the day when CRP decreased to <1.0 mg/dL on at least 2 consecutive measurements, the WBC count returned to the normal range (4–10 × 10^3^/μL), and clinical symptoms subsided. According to these criteria, a total of 10 infectious periods were identified: Episode 1 (from November 28, 2022, to January 15, 2023), Episode 2 (from February 5 to July 5, 2023), Episode 3 (from July 31 to August 6, 2023), Episode 4 (from November 5 to November 15, 2023), Episode 5 (from January 1 to January 24, 2024), Episode 6 (from March 6 to March 21, 2024), Episode 7 (from June 14 to August 11, 2024), Episode 8 (from August 28 to September 8, 2024), Episode 9 (from February 24 to March 11, 2025), and Episode 10 (from May 4 to June 6, 2025).

To quantify systemic inflammatory response more comprehensively than through assessment of CRP levels or WBC counts alone, 2 hematological markers, NLR and SIRI, were assessed. NLR was adopted as a validated marker of systemic inflammation, with a reported reference range of 0.78 to 3.53 in normal adults.^[15,16]^ SIRI was first proposed by Qi et al.^[17]^ and has since been validated in various clinical contexts, including advanced cancer and stroke.^[15,16,18]^ Recent evidence suggests that SIRI may outperform NLR in reflecting systemic inflammation and predicting adverse outcomes.^[16,18]^ Although well-established normal reference values for SIRI remain lacking, prior research has suggested that SIRI values >1.4 are associated with an increased risk of all-cause and cardiovascular mortality in general population cohorts.^[12]^ In specific disease contexts, such as chronic liver disease, SIRI thresholds of approximately 2.0 have shown prognostic relevance for short-term mortality.^[19]^

The markers were calculated using absolute cell counts:

- NLR = neutrophils/ lymphocytes
- SIRI = neutrophils × monocytes/ lymphocytes

When only differential percentages were available, absolute counts were derived using the total WBC counts.

These markers were evaluated during each infectious period and longitudinally throughout the follow-up period. This approach enabled assessment of their correlation with the clinical course and outcomes. It also provided an objective framework for monitoring systemic inflammation and evaluating treatment responses to integrative therapy, including the effectiveness of concurrent herbal medications during infectious periods.

To assess the safety of long-term IPMC, liver function tests (LFTs) and renal function tests (RFTs) were performed. Liver function was assessed based on AST, ALT, and GGT levels. The upper limit of normal (ULN) for AST and ALT levels was set to 40 IU/L based on previous studies,^[20]^ and the ULN for GGT levels was set to 64 IU/L according to the reference values from Kyung Hee University Hospital.

Liver injury, especially drug-induced liver injury (DILI), was defined as ALT levels exceeding 5 times the ULN or GGT exceeding 3 times the ULN, according to the Council for International Organizations of Medical Sciences laboratory criteria and Guidelines for the Management of Cholestatic Liver Diseases (2021). To assess the safety of IPMC2 during infectious periods, the mean and standard deviation of LFT results were calculated for the periods of herbal medication monotherapy versus IPMC2, and the 2 were compared. The number of days with antibiotic monotherapy was limited (eight in the 2nd infectious period and one in the 3rd infectious period); therefore, LFTs during antibiotic monotherapy were not included to focus on the safety evaluation of herbal medications. For noninfectious periods, no comparison group was formed because the patients received herbal medications along with other medications for managing brainstem hemorrhage, tremor/spasticity, hypertension, dyslipidemia, or upper gastrointestinal (UGI) bleeding throughout noninfectious periods. Therefore, the LFT results were analyzed to evaluate the safety of IPMC1.

Renal function was examined over the entire study period using BUN levels, SCr levels, and modification of diet in renal disease-estimated glomerular filtration rate. The ULNs for BUN and SCr levels were set to 20 mg/dL and 0.95 mg/dL, respectively, based on Kyung Hee University Hospital reference values. The lower limit of normal for eGFR was set to 90 mL/minute/1.73 m^2^ according to previous studies.^[21]^

Several diagnostic challenges were encountered during patient assessment and follow-up. First, the patient’s tracheostomy status precluded verbal communication, which substantially limited the accurate evaluation of consciousness and cognitive impairment. Consequently, assessments relying on verbal responses, such as the verbal components of the GCS and K-MMSE, could not be reliably performed. Second, assessing renal function is challenging. Severe muscle atrophy resulting from quadriplegia led to artificially low SCr levels. This, in turn, caused a substantial overestimation of eGFR, making it an unreliable marker for monitoring true renal function in this patient. No other financial-, cultural-, or language-related barriers impeded the diagnostic process.

### 2.4. Biomedical care

#### 2.4.1. Conventional treatment

IPMC1. A collaborative approach with the neurology department was implemented to manage brainstem hemorrhage. Lacosamide (Vimsk tablets) were prescribed at a dosage of 150 mg twice daily from November 23, 2022, to June 30, 2025. Clonazepam (Rivotril tablet) was prescribed according to rigidity at a dosage of 0.5 mg twice daily from May 24, 2024, to June 30, 2025. Baclofen (Baclofen tablets) were prescribed simultaneously at a dosage of 20 mg twice daily. Additionally, physical therapy was implemented, comprising 30 minutes of rehabilitative development therapy for central nervous system disorders and 30 minutes of gait training.

IPMC2. Antimicrobial agents are prescribed to manage infections such as UTI, pneumonia, and COVID-19. During the 1st infectious episode, an antifungal agent (fluconazole) and a cephalosporin antibiotic (ceftriaxone) were administered. In the 2nd infectious episode, a broader spectrum of drugs was administered, including cephalosporins (cefepime and ceftriaxone), a glycopeptide (vancomycin), carbapenems (meropenem and ertapenem), fluoroquinolones (moxifloxacin and levofloxacin), and an antifungal agent (fluconazole). Antimicrobial agents were not administered during the 3rd infectious episode. Treatment resumed in the 4th infectious episode, with carbapenems (ertapenem and meropenem). This was followed by the use of a carbapenem (ertapenem) and glycopeptide (teicoplanin) in the 5th infectious episode. For a COVID-19 infection in the 6th infectious episode, an antiviral agent (remdesivir) and a cephalosporin (ceftriaxone) were administered. A cephalosporin (ceftazidime) was used alone during 2 separate periods in the 7th infectious episode, and again in the 8th infectious episode. In the 9th infectious episode, a carbapenem (meropenem) was combined with a cephalosporin (ceftazidime). Finally, in the 10th infectious episode, cephalosporins (cefepime and ceftazidime) and a fluoroquinolone (levofloxacin) were prescribed.

Notably, the patient required multiple courses of broad-spectrum antibiotics, including meropenem and vancomycin, to manage recurrent and severe infections during the 2nd, 4th, and 9th infectious episodes.

IPMC3. For managing hypertension, amlodipine 5 mg/olmesartan 20 mg (Sevikar tablets) were prescribed at a dosage of 2 tablets (T) once daily from November 23, 2022, to December 27, 2022. For managing dyslipidemia, ezetimibe 10 mg/atorvastatin 10 mg (Atozet tablets) were administered once daily from December 12, 2022, to April 05, 2023, based on an abnormal lipid profile. Subsequently, pravastatin and fenofibrate (Pravafenix capsules) were administered from September 04, 2024, to November 07, 2024. During this period, ezetimibe 10 mg (Ezetrol tablets) were prescribed from October 10, 2024, to November 07, 2024. The treatment was later switched to rosuvastatin 10 mg (Rosulord tablets) and ezetimibe, fenofibrate (Ezefeno tablets), both administered from November 06, 2024. Rosulord prescription was stopped on December 04, 2024, and replaced by rosuvastatin 20 mg (Crestor) starting December 05, 2024, which continued alongside Ezefeno until June 30, 2025.

#### 2.4.2. Personalized acupuncture therapies

An acupuncture regimen was developed based on the traditional acupuncture theory of meridians. To manage hemorrhagic stroke, acupuncture was administered at the “seven acupoints for stroke,” comprising GV20, GB7, LI15, GB31, LI11, GB39, and ST36, which have been extensively investigated for their therapeutic efficacy in cerebrovascular disorders. Additionally, based on the patient’s So-Yang constitutional type, Saam acupuncture was administered to acupoints LU8, KI7, SP3, and KI3 (bilaterally) to strengthen kidney function and manage stroke. A total of 21 acupoints were stimulated in each session. The patient received daily 20-minute acupuncture sessions throughout hospitalization.

All procedures were performed by a skilled traditional Korean medicine doctor using single-use stainless steel needles (Dongbang Acupuncture Inc., Korea, 0.20 mm × 30 mm) inserted to a depth of 10 to 20 mm. The needles were retained for 20 minutes, with electrical stimulation at 2 Hz applied to acupoints LI4, LI11, LR3, and ST36. Detailed information on the acupuncture regimen, administered in accordance with the STRICTA guidelines is summarized in Table 1.

**Table 1:** Acupuncture therapy administered tot he patient for managing brainstem haemorrhage.

1. Details of needling	(1) Number of needle insertions per subject per session: 20 (2) Acupuncture method: based on the 7 acupoints for stroke and kidney tonification of Saam acupuncture, we administered acupuncture according to the patient’s past history of stroke and constitutional type (So-Yang type) (3) Acupoints used: Baihui (GV20), Qubin (GB7), Jianyu (LI15), Fengshi (GB31), Quchi (LI11), Xuanzhong (GB39), Zusanli (ST36), Jingqu (LU8), Fuliu (KI7), Taibai (SP3), and Taixi (KI3), bilateral (4) Depth of insertion: 10–20 mm (5) Response sought: Simple insertion (6) Needle stimulation: Manual (7) Needle retention time: 20 minutes (8) Needle type: Single-use acupuncture needles (0.20 mm × 30 mm stainless steel)
2. Treatment regimen	(1) Number of treatment sessions: 938 (2) Frequency and duration: Once daily during hospitalization
3. Other components of treatment	(1) Other interventions: Electrical stimulation (2 Hz, 20 minutes) applied to bilateral Hegu (LI4), Quchi (LI11), Taichong (LR3), and Zusanli (ST36) (2) Setting: Kyung Hee University Korean Medicine Hospital
4. Practitioner background	Licensed TKM doctor
5. Control or comparator intervention	*Not applicable*

CV = conception vessel, GB = gall bladder, GV = governor vessel, KI = kidney, LI = large intestine, LR = liver, LU = lung, SP = spleen, ST = stomach, TKM = traditional Korean medicine.

#### 2.4.3. Personalized herbal medication therapies

Throughout the treatment period, 9 herbal medications were administered, including 6 for managing brainstem hemorrhage and 3 for controlling inflammation. Considering the patient’s overall condition, personalized herbal medicines were administered as the main treatment throughout hospitalization.

For managing brainstem hemorrhage, the long-term IPMC1 included administration of Hyeongbangdojeok-san, Hyeongbangsabaek-san, Yanggyeoksanhwa-tang, Hyeongbangjihwang-tang, Dokhwaljihwang-tang, and Sipimijihwang-tang. For treating infections, the long-term IPMC2 included administration of Jeoryeongchajeonja-tang, Jihwangbaekho-tang (JHBT), and Jyeoryeongbaekho-tang. During infectious periods, treatments were selected based on the specific diagnosis: Jeoryeongchajeonja-tang was prescribed for UTIs, JHBT for pneumonia, and Jyeoryeongbaekho-tang for concurrent UTI and pneumonia or inaccurate infections. All herbal medicines were prepared by boiling them in 1000 mL of distilled water for approximately 2 hours until the volume of the solution was reduced to 100 mL. All decoctions were prescribed in 2 daily doses, with the patient consuming 100 mL 3 times a day. The composition and dosage of each prescription are detailed in Table 2.

**Table 2:** Compositions and functions of herbal medicines prescribed for the patient.

Herb	Brainstem hemorrhage treatment						Infection treatment			Effect in TKM
	HDS	HSS	YST	HJT	DJT	SJT	JCT	JHBT	JRBT	
*Osterici Radix* (Ganghwal)	4	4		4			4			Exterior-releasing
*Schizonepetae Spica* (Hyeonggae)	4	4	4	4		4	4			
*Saposhnikovia Radix* (Bangpung)	4	4	4	4	4	4	4	4		
*Angelicae Pubescentis Radix* (Dokhwal)	4	4		4	4		4	4		
*Rehmannia glutinosa Libosch* (Saengjihwang)	12	12	12		8			16	16	Stomach heat-clearing
*Gypsum Fibrosum* (Seokgo)		4	12		8		4	20	16	
*Anemarrhenae Rhizoma* (Jimo)		4	4				4	8	14	
*Polyporus* (Jyeoryung)							6		4	Bladder heat-clearing Urination-promoting
*Plantaginis Semen* (Chajeonja)				4		4	6			
*Phellodendri Cortex* (Hwangbaek)									4	
*Hoelen* (Baekbokryeong)	4	8		8	6	6	8			Urination-promoting
*Alismatis Rhizoma* (Taeksa)	4	8		8	6	6	8		4	
*Lycii Radicis Cortex* (Jigolpi)						4				Stagnation-resolving Head–eye clearing
*Lonicerae Folium et Caulis* (Indong)			8							
*Forsythiae Fructus* (Yeongyo)			8							
*Gardeniae Fructus* (Chija)			4							
*Menthae Herba* (Bakha)			4							
*Scrophulariae Radix* (Hyeonsam)	6					4				Phlegm-dispelling
*Trichosanthis Semen* (Gualuin)	6									
*Peucedani Radix* (Jeonho)	4									
*Rehmanniae Radix Preparata* (Sukjihwang)				8	16	16				Kidney-tonifying
*Corni Fructus* (Sansuyu)				8	8	8				
*Rubi Fructus* (Bokbunja)						4				
*Lycii Fructus (Gugija*)						4				

DJT = Dokhwaljihwang-tang, HDS = Hyeongbangdojeok-san, HJT = Hyeongbangjihwang-tang, HSS = Hyeongbangsabaek-san, JCT = Jeoryeongchajeonja-tang, JHBT = Jihwangbaekho-tang, JRBT = Jyeoryeongbaekho-tang, SJT = Sipimijihwang-tang, TKM = traditional Korean medicine, YST = Yanggyeoksanhwa-tang.

## 3. Results

### 3.1. Efficacy of long-term IPMC implemented over 2 years for managing brainstem hemorrhage

#### 3.1.1. Functional outcomes

Regarding neurological outcomes, MBI scores (shown in Fig. 3A) consistently remained 0 from February 2, 2024, to July 1, 2025, indicating total dependence in activities of daily living. K-MMSE scores (shown in Fig. 3B) consistently remained 0 from January 12, 2023, to July 1, 2025, indicating severe cognitive impairment due to brainstem hemorrhage. GDS scores (shown in Fig. 3C) ranged between 6 and 7, indicating severe cognitive impairment. GCS scores (shown in Fig. 3D) consistently remained at 11, with a slight increase to 12 observed on June 29, 2023. LOC (shown in Fig. 3E) was maintained at Grade II (drowsy), with an improvement to Grade I (alert) observed on July 20, 2023.

**Figure 3.**
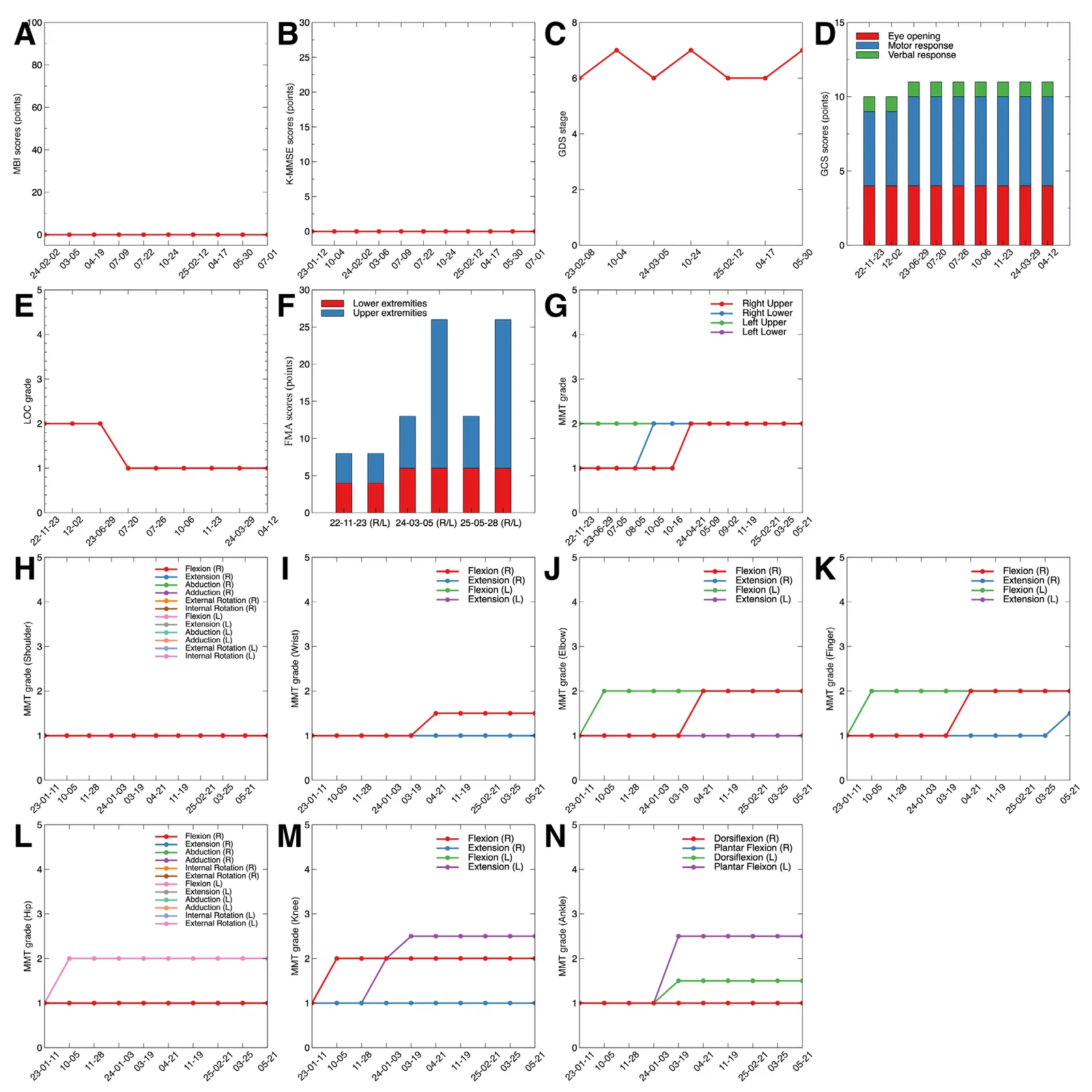
Functional outcomes. (A) MBI scores (points); (B) K-MMSE scores (points); (C) GDS stage; (D) GCS scores (points); (E) LOC grade; (F) FMA scores (points); (G) MMT grade; (H) MMT grade (shoulder); (I) MMT grade (wrist); (J) MMT grade (elbow); (K) MMT grade (finger); (L) MMT grade (hip); (M) MMT grade (knee); (N) MMT grade (ankle). Upper and lower limb MMT scores were recorded based on the MMT 5-point scale, and joint-specific MMT scores were recorded based on the modified MMT scale. For graphical representation, modified MMT scale scores of 2+, 2, and 2 were recorded as 2.5, 2, and 1.5, respectively. FMA = Fugl-Meyer assessment, GCS = Glasgow Coma Scale, GDS = global deterioration scale, K-MMSE = Korean mini-mental state examination, L = left, LOC = level of consciousness, MBI = modified Barthel index, MMT = manual muscle testing, R = right.

Regarding motor outcomes, FMA scores (shown in Fig. 3F) were 8-13-13 on the right side and 8-26-26 on the left side, indicating an overall improvement on both sides. Recovery was faster on the left side than on the right side. MMT grades (shown in Fig. 3G) for both upper and lower extremities improved from Grade 1 to Grade 2, with faster recovery on the left side. Joint-specific MMT (shown in Fig. 3H-N) demonstrated an overall progression from Grade 1 to Grade 2. Some joints, such as the knee and ankle, showed improvements up to Grade 2+. Recovery was faster in the lower extremities than in the upper extremities and more prominent in the distal joints (e.g., wrist, finger, ankle) than in the proximal joints (e.g., hip and shoulder).

#### 3.1.2. Inflammation management related to complications

Inflammatory markers demonstrated distinct differences between infectious and noninfectious periods. NLR increased more during infectious periods (mean 0.57) than during noninfectious periods (mean 0.29) (shown in Fig. 4A). Notably, values remained within the normal range (0.78–3.53) throughout the observation period and never exceeded the upper limit (3.53) even during infectious periods. SIRI values were more elevated during the infectious periods (mean 2.83, peaks > 12) than during the noninfectious periods (mean 1.74) (shown in Fig. 4B). Longitudinal trends in CRP levels further demonstrated recurrent spikes above 1.0 to 2.0 mg/dL, consistent with infectious episodes, consistent with combined clinical and laboratory criteria, whereas levels mostly remained < 1.0 mg/dL during noninfectious periods (shown in Fig. 4C).

**Figure 4.**
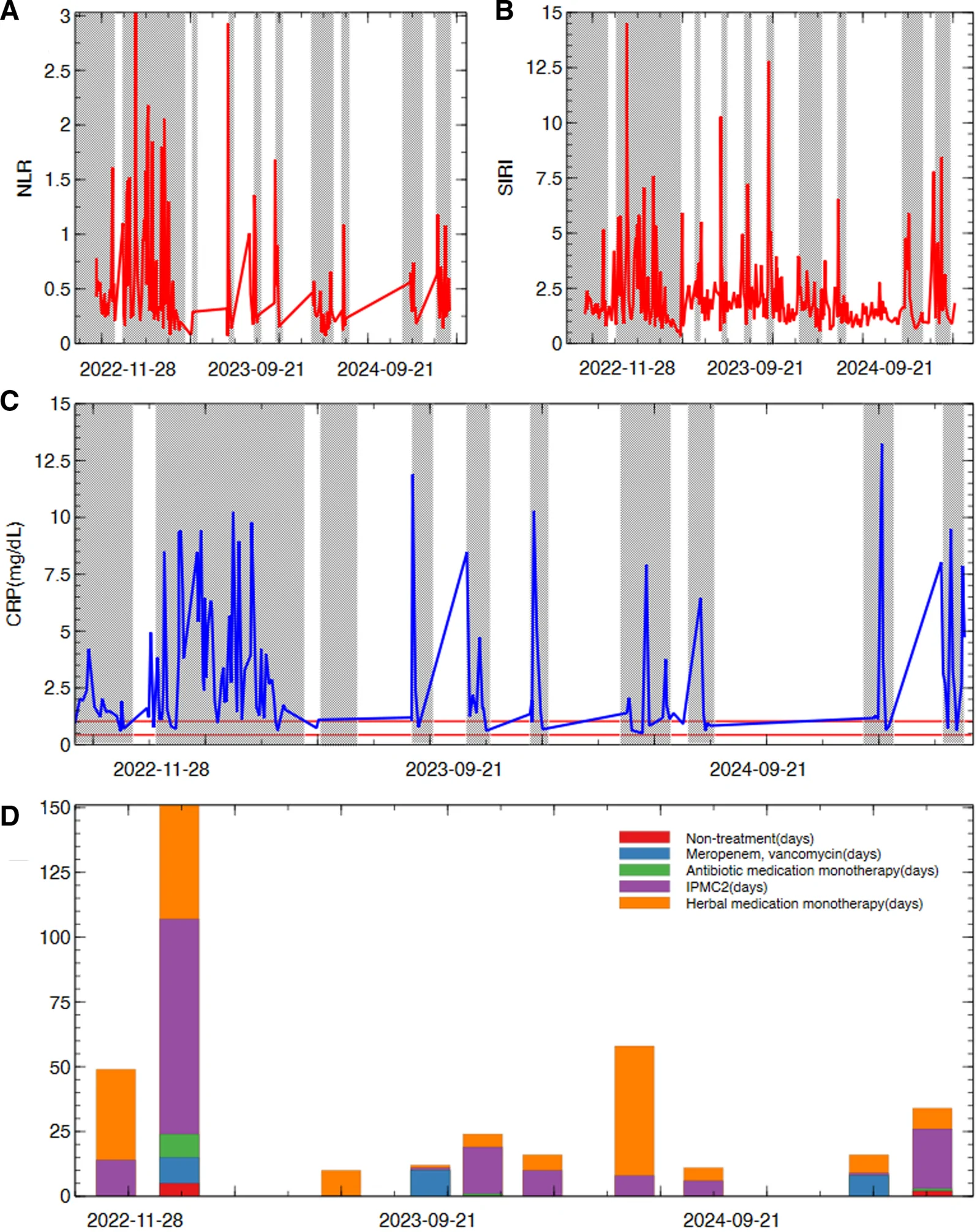
Infection-related indicators. (A) NLR; (B) SIRI; (C) CPR (mg/dL); (D) patterns of antimicrobial and herbal medication use. Gray shading indicates infectious periods. Numbers under the X-axis indicate dates at admission and at the first and second years after onset. The ULNs for NLR and CRP levels are 0.78 to 3.53 mg/dL and <0.5 mg/dL, respectively. A consensus ULN for SIRI has not been established. Red lines on the CRP graph represent 0.5 mg/dL and 1.0 mg/dL, respectively. Each bar in the bar chart represents an infectious period (1st–10th). 1st infectious period: November 28, 2022, to January 15, 2023. 2nd infectious period: February 5, 2023, to July 5, 2023. 3rd infectious period: July 31, 2023, to August 6, 2023. 4th infectious period: November 5, 2023, to November 15, 2023. 5th infectious period: January 1, 2024, to January 24, 2024. 6th infectious period: March 6, 2024, to March 21, 2024. 7th infectious period: June 14, 2024, to August 11, 2024. 8th infectious period: August 28, 2024, to September 8, 2024. 9th infectious period: February 24, 2025, to March 11, 2025. 10th infectious period: May 4, 2025, to June 6, 2025. CRP = C-reactive protein, IPMC = integrative personalized medicine care, NLR = neutrophil-to-lymphocyte ratio, SIRI = systemic inflammation response index, ULN = upper limit of normal.

The patient met the criteria for meropenem and vancomycin administration during the 2nd, 4th, and 9th infectious periods. Antibiotic or antifungal medication monotherapy was administered more frequently in the early course, including 9 days during the 2nd episode and shorter courses thereafter. According to the progress notes, the total number of days of antibiotic use was 66 in the first year and 16 in the second year. Notably, broad-spectrum antibiotics, such as meropenem and vancomycin, accounted for only a limited fraction of the overall course, reflecting their restricted use (shown in Fig. 4D).

Herbal medication prescriptions were consistently integrated throughout the clinical course, either in combination with antibiotics or as monotherapy. Herbal medication monotherapy was predominantly administered during the 1st (35 of 49 days), 3rd (10 of 10 days), and 7th (10 of 12 days) infectious periods. Over time, the proportion of periods covered by herbal medications alone or in combination with general antibiotics increased, whereas reliance on broad-spectrum antibiotics declined (shown in Fig. 4D).

### 3.2. Safety of long-term IPMC implemented over 2 years for managing brainstem hemorrhage

#### 3.2.1. Liver function

Overall, AST, ALT, and GGT levels remained below the ULN (shown in Fig. 5A). As shown in Table 3, the number of measurements exceeding the ULN was 21 (8.7%) for AST, 41 (14.7%) for ALT, and 49 (15.7%) for GGT. Episodes meeting the criteria for DILI occurred 6 times for ALT and 5 times for GGT. However, these liver function abnormalities were transient and resolved within a week.

**Table 3:** Liver and renal function test results.

Parameter	Reference range	Abnormal cases, n/N (%)	DILI (n)
AST	ULN = 40 U/L	Above ULN: 21/310 (6.8%)	*NA*
ALT	ULN = 40 U/L	Above ULN: 41/311 (13.2%)	5*ULN = 6
γ-GGT	ULN = 64 U/L	Above ULN: 48/311 (15.4%)	3*ULN = 5
BUN	ULN = 20 mg/dL	Above ULN: 5/310 (1.61%)	*NA*
SCr	ULN = 0.95 mg/dL	Above ULN: 0/309 (0%)	*NA*
eGFR	LLN = 90 mL/min/1.73m^2^	Below LLN: 1/309 (0.3%)	*NA*

Values are presented as n/N (%) (number of days above ULN or below LLN/ total number of days (percentage of total days meeting the criteria)] or n (number of days meeting the criteria for DILI). Abnormal cases were defined as values above the ULN (for AST, ALT, γ-GGT, BUN, sCr) or below the LLN (for eGFR). DILI was defined according to CIOMS criteria (ALT more than 5 times the ULN) and guidelines for the management of cholestatic liver diseases (γ-GGT more than 3 times the ULN).

ALT = alanine aminotransferase, AST = aspartate aminotransferase, BUN = blood urea nitrogen, CIOMS = Council for International Organizations of Medical Sciences, DILI = drug-induced liver injury, eGFR = estimated glomerular filtration rate, LLN = lower limit of normal, SCr = serum creatinine, ULN = upper limit of normal, γ-GGT = gamma-glutamyl transferase.

**Figure 5.**
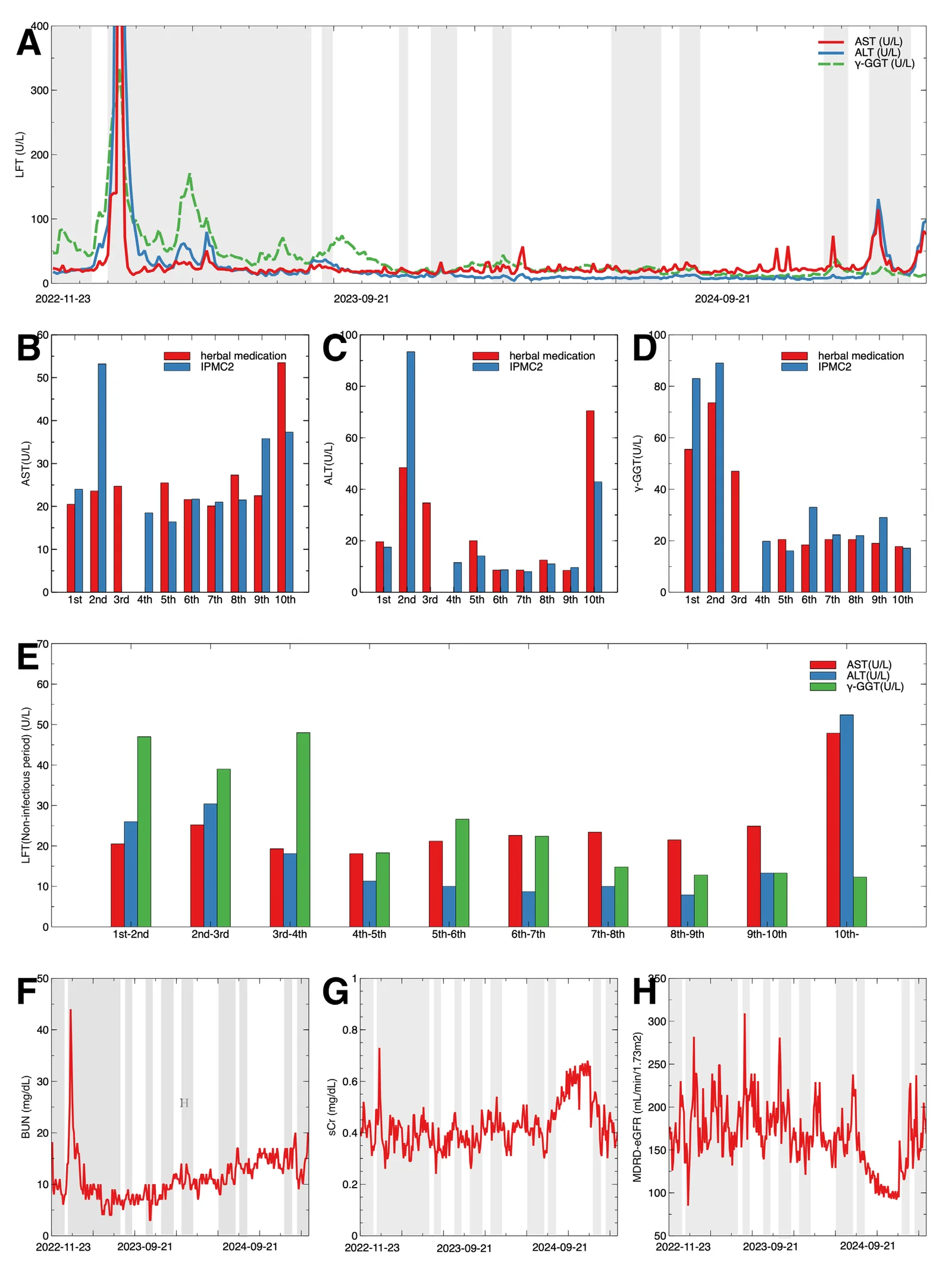
Liver and renal function test results. (A) LFT (U/L); (B) AST (U/L); (C) ALT (U/L); (D) γ-GGT (U/L); (E) LFT (non-infectious period) (U/L); (F) BUN (mg/dL); (G) sCr (mg/dL); (H) MDRD-eGFR (mL/min/1.73 m^2^). Gray shading indicates infectious periods. Numbers under the X-axis indicate dates at admission and at the first and second years after onset. The ULNs for BUN, SCr, AST, ALT, and GGT levels are 20 mg/dL, 0.95 mg/dL, 40 U/L, 40 U/L, and 64 U/L, respectively. The LLN for eGFR is 90 mL/min/1.73 m^2^. 1st infectious period: November 28, 2022, to January 15, 2023. 2nd infectious period: February 5, 2023, to July 5, 2023. 3rd infectious period: July 31, 2023, to August 6, 2023. 4th infectious period: November 5, 2023, to November 15, 2023. 5th infectious period: January 1, 2024, to January 24, 2024. 6th infectious period: March 6, 2024, to March 21, 2024. 7th infectious period: June 14, 2024, to August 11, 2024. 8th infectious period: August 28, 2024, to September 8, 2024. 9th infectious period: February 24, 2025, to March 11, 2025. 10th infectious period: May 4, 2025, to June 6, 2025. ALT = alanine aminotransferase, AST = aspartate aminotransferase, BUN = blood urea nitrogen, GGT = γ-glutamyltransferase, LFTs = liver function tests, LLN = lower limit of normal, MDRD-eGFR = modification of diet in renal disease-estimated glomerular filtration rate, RFTs = renal function tests, SCr = serum creatinine, ULN = upper limit of normal.

During infectious periods, AST, ALT, and GGT levels remained below the ULN (shown in Fig. 5A). Differences in LFT results between herbal medication monotherapy and IPMC2 were negligible (shown in Fig. 5B-D). The details are presented in Table 4. Transient elevations in AST, ALT, and GGT levels were noted in IPMC2 during the 2nd infectious period (from February 5, 2023, to July 5, 2023) and in herbal medication monotherapy during the 10th infectious period (from May 4, 2025, to June 6, 2025).

**Table 4:** Changes in liver function test results across infectious episodes.

	LFT	1st	2nd	3rd	4th	5th	6th	7th	8th	9th	10th
IPMC2	AST	24 ± 5.7	53.2 ± 142.8	*NA*	18.5 ± 4	16.4 ± 1.7	21.7 ± 5.9	21 ± 6	21.5 ± 0.7	35.8 ± 21.4	37.3 ± 21.5
	ALT	17.5 ± 0.7	93.4 ± 207.1	*NA*	11.5 ± 1	14.1 ± 3	8.7 ± 2.5	8 ± 1	11	9.6 ± 1.9	42.9 ± 25.6
	γ-GGT	83 ± 1.4	89 ± 73.6	*NA*	19.8 ± 1	16.1 ± 1.8	33 ± 3	22.3 ± 4.2	22	29 ± 6.4	17.1 ± 3.3
Herbal medication,monotherapy	AST	20.5 ± 1.6	23.6 ± 8.3	24.7 ± 1.2	*NA*	25.5 ± 10.6	21.6 ± 6.8	20.1 ± 3.1	27.3 ± 4.6	22.5 ± 0.7	53.5 ± 45.2
	ALT	19.6 ± 2.5	48.4 ± 35.9	34.7 ± 2.1	*NA*	20 ± 7	8.6 ± 3.3	8.6 ± 0.7	12.5 ± 0.6	8.5 ± 0.7	70.5 ± 55.9
	γ-GGT	55.5 ± 10.7	73.6 ± 37.3	47 ± 2.6	*NA*	20.5 ± 0.7	18.4 ± 7.2	21.6 ± 3.5	20.5 ± 2.6	19 ± 1.4	17.75 ± 5.5

Infectious periods were determined based on an elevation in CRP levels and clinical findings.

1st infectious period: November 28, 2022, to January 15, 2023.

2nd infectious period: February 5, 2023, to July 5, 2023.

3rd infectious period: July 31, 2023, to August 6, 2023.

4th infectious period: November 5, 2023, to November 15, 2023.

5th infectious period: January 1, 2024, to January 24, 2024.

6th infectious period: March 6, 2024, to March 21, 2024.

7th infectious period: June 14, 2024, to August 11, 2024.

8th infectious period: August 28, 2024, to September 8, 2024.

9th infectious period: February 24, 2025, to March 11, 2025.

10th infectious period: May 4, 2025, to June 6, 2025.

IPMC2 was used to manage infections (urinary tract infections, pneumonia, and COVID-19).

Values are presented as mean ± standard deviation.

ALT = alanine aminotransferase, AST = aspartate aminotransferase, IPMC = integrative personalized medicine care, γ-GGT = gamma-glutamyl transferase.

During noninfectious periods, AST, ALT, and GGT levels remained below the ULN (shown in Fig. 5A). Isolated elevations in GGT levels were observed between the 1st and 2nd infectious periods (from January 16, 2023, to February 4, 2023) and between the 3rd and 4th infectious periods (from August 7, 2023, to November 4, 2023). Additionally, elevations in AST and ALT levels were observed after the 10th infectious period (from June 7, 2025) (shown in Fig. 5E).

#### 3.2.2. Renal function

As shown in Fig. 5F-H, during infectious and noninfectious periods, BUN and SCr values generally remained below the ULN. Furthermore, eGFR values were maintained above the lower limit of normal during both periods. Across the entire observation period, deviations from the normal range occurred 5 times (1.61%) for BUN, 0 time (0%) for SCr, and 1 time (0.3%) for eGFR (shown in Table 3). All these events occurred during the early phase of the 2nd infectious period (from February 5, 2023, to July 5, 2023).

The patient adhered to the prescribed medications and therapy. Although no subjective discomfort or severe adverse effects were reported, transient elevations in liver function markers (AST, ALT, and GGT) and renal function markers (BUN) were observed on several occasions, as detailed in the safety analysis section.

## 4. Discussion

### 4.1. Efficacy of long-term IPMC implemented over 2 years for managing brainstem hemorrhage

#### 4.1.1. Functional outcomes

The patient’s K-MMSE score remained at 0, and GDS scores were 6 to 7, indicating severe cognitive impairment. However, considering that the patient had a tracheostomy tube in place and verbal communication was impossible, these measures may not accurately reflect cognitive function. GCS and LOC scores were assessed based on clinical observations. GCS scores improved slightly from 11 to 12. The eye opening (E) and motor response (M) components had maximum scores, whereas the verbal response (V) component remained at 1 because of the tracheostomy tube. Therefore, unless the tracheostomy tube is removed, the GCS score is expected to remain unchanged, suggesting that a patient’s LOC can be considered near-optimal. Specifically, at the time of admission, the patient could only blink on command. By July 2025, the patient could follow simple commands, raise a hand, or nod for communication.

MBI scores remained at 0, reflecting the total level of assistance. Since the MBI primarily assesses independence in activities of daily living, this suggests only minimal functional improvement. In contrast, motor outcomes measured using the MMT and FMA demonstrated notable improvements in limb motor function. At the time of admission, the patient was unresponsive to commands and unable to perform limb movements or elevation. By July 2025, finger movement, knee flexion and extension, and left lower limb elevation were possible, and the grasping function had returned. Overall, motor recovery was faster on the left side than on the right side, in the lower limbs than in the upper limbs, and in the distal joints than in the proximal joints, consistent with clinical observations.

Previous studies on brainstem hemorrhage and stroke recovery have reported that motor function of the lower limbs often recovers faster than that of the upper limbs, consistent with the central pattern generators (CPGs) hypothesis. Recovery of trunk control and motor and sensory functions of the lower limbs occurred rapidly, within 3 months after onset. Function of upper limbs and gait improves significantly from 3 to 6 months lasting up to 12 months after onset.^[22]^ Additionally, lateralized lesions can lead to asymmetrical recovery between the left and right sides, as suggested by corticospinal tract (CST) preservation patterns.^[23]^ While proximal joint movements usually recover faster than distal movements, selective distal joint recovery has been reported depending on lesion location and the neural circuits involved.^[24]^ These findings provide context for interpreting the atypical recovery patterns observed in this patient.

In this patient, faster recovery of the lower limbs than that of the upper limbs can be explained by the CPGs hypothesis. CPGs, located predominantly in the lumbar spinal cord, generate rhythmic motor patterns, even without input from higher motor centers. Because these networks primarily innervate the lower limbs, recovery of the lower limbs is faster than that of the upper limbs.^[22]^ The difference in recovery between the left and right sides can be explained by the CST. The hemorrhage was slightly lateralized to the left side. Consequently, the left-sided CST may have been relatively preserved compared to the right, thus the left side recovered faster than the right side.^[25]^ Faster recovery of distal joints can be attributed to the combination of lesion location, neuroplasticity, and targeted rehabilitation interventions. These neural and therapeutic factors collectively explain the observed joint- and side-specific recovery patterns.

To improve the functional outcomes, herbal medications that were designed to enhance the patient’s general condition, guided by the principles of Sasang constitutional medicine, were prescribed. Experimental research has demonstrated that key medicinal herbs commonly found in administered formulations possess significant neuroprotective properties that contribute to functional rehabilitation of patients with hemorrhagic stroke. Studies have shown that *Rehmannia glutinosa Libosch* (Saengjihwang), as a main component of Sheng-Di-Da-Huang Decoction, significantly improved neurological function scores and reduced brain water content in rats with intracerebral hemorrhage by inhibiting inflammation and protecting blood–brain barrier integrity.^[26]^ *Hoelen* (Baekbokryeong) extract has been demonstrated to provide robust protection against tissue plasminogen activator-induced hemorrhagic transformation after stroke by modulating microglial M1/M2 phenotype polarization and regulating the IRF5-IRF4 axis, thereby reducing pro-inflammatory cytokine secretion and enhancing anti-inflammatory responses and thereby allowing neuroprotection.^[27]^ Therefore, the strategic use of these herbal medicines was implemented to optimize the patient’s functional recovery through their established neuroprotective mechanisms and neuroplasticity-enhancing properties.

Additionally, acupuncture was performed to enhance functional outcomes. When combined with conventional rehabilitation, acupuncture is known to be beneficial for improving post-stroke sequelae, such as paralysis, spasticity, dysphagia, and cognitive impairment.^[5–7]^ Notably, clinical studies have demonstrated that acupuncture combined with rehabilitation leads to significant improvements in muscle strength, as measured by the MMT scale.^[28]^ Given this evidence, it is plausible that the marked improvement in MMT scores in this patient can be, at least in part, attributed to the inclusion of acupuncture in the treatment regimen.

#### 4.1.2. Inflammation management related to complications

This case highlights the evolving dynamics of inflammation management in recurrent infections. By examining the temporal patterns of CRP levels, NLR, and SIRI, we monitored changes in inflammatory markers during infectious periods under sustained herbal medications. In this case, CRP levels fluctuated between 0.5 and 11.9 mg/dL, while NLR increased more modestly during infectious periods (mean 0.57) than during noninfectious periods (mean 0.29). SIRI values showed a similar pattern, averaging 2.83 during infectious periods and 1.74 during noninfectious periods, highlighting its role as an adjunctive marker of systemic inflammation. Moreover, the integration of SIRI into longitudinal monitoring further strengthened the ability to contextualize inflammatory activity, underscoring its potential as an adjunctive biomarker for guiding therapeutic decisions. Collectively, these markers provided complementary perspectives.

A notable finding in this case was that inflammation control with herbal medications alone was feasible when CRP levels remained below 2.0 mg/dL. Prolonged antibiotic exposure is associated with adverse outcomes such as antimicrobial resistance, gut microbiota dysbiosis, nephrotoxicity, and *Clostridioides difficile* infection.^[29–31]^ However, during infectious periods, the patient’s clinical symptoms were stably managed, and recurrence did not necessitate additional antibiotic coverage. This observation is consistent with the findings of previous studies reporting that CRP cutoff values can be used as reliable thresholds for guiding antibiotic discontinuation in infectious diseases.^[4,32]^ However, it is important to consider whether a part of the observed inflammation control during the herbal medication monotherapy phases could have been influenced by residual post-antibiotic effects (PAEs). Vancomycin has been shown to exert measurable PAEs against methicillin-resistant *Staphylococcus aureus* strains, typically suppressing bacterial regrowth for several hours after the concentrations fall below the minimum inhibitory concentration (MIC).^[33]^ Similarly, meropenem has demonstrated PAEs against Enterobacteriaceae, with durations generally lasting for a few hours depending on the assessment method.^[34]^ In addition, in vitro studies have shown that ceftriaxone monotherapy exhibits a PAE lasting up to 145 minutes at the MIC and 50 minutes at one-half the MIC.^[35]^ These findings indicate that a transient suppressive effect may persist shortly after discontinuation of broad-spectrum agents. However, in the present case, inflammation control observed during herbal medication monotherapy extended over multiple days to weeks, with CRP levels, NLR, and SIRI remaining stable below the critical thresholds. Although certain studies have reported longer PAE durations under specific conditions, these typically do not extend beyond a day,^[33,34]^ and thus cannot fully explain the multiday inflammation control observed in this case. Our findings extend this evidence by demonstrating that antibiotics can be safely withheld at low CRP levels and inflammation may be effectively managed through sustained herbal medications alone.

During the early course and selected later periods (notably the 9th), broad-spectrum antibiotics, such as meropenem and vancomycin, were required to control systemic inflammation. In this case, their use was prompted by a recurrent infection presenting with high fever and a systemic inflammatory response. However, the reliance on such antibiotics diminished over time, as the cumulative days of antibiotic use steadily decreased over 2 years according to progress notes. Herbal medications played a progressively central role in this transition. For example, JHBT derivatives were frequently prescribed to control febrile and inflammatory states, supported by experimental evidence of JHBT’s anti-inflammatory activity in dermatitis and fever models.^[36]^ Notably, inflammation control with herbal medications alone was observed during the 3rd infectious period, when no antibiotics were administered. In the 1st, 6th, 7th, and 8th infectious periods, IPMC or herbal medication monotherapy was used, indicating a treatment approach centered on herbal medications.

### 4.2. Safety of long-term IPMC implemented over 2 years for managing brainstem hemorrhage

#### 4.2.1. Liver function

According to the Council for International Organizations of Medical Sciences laboratory criteria, DILI is defined as ALT more than 5 times the ULN, alkaline phosphatase more than 2 times the ULN, or ALT more than 3 times the ULN with total bilirubin more than 2 times the ULN. The 2021 Guidelines for the Management of Cholestatic Liver Diseases additionally include GGT more than 3 times the ULN as a criterion. In this patient, sudden fluctuations in LFT results were analyzed according to these standards.

Overall, during the entire observation period, AST, ALT, and GGT levels exceeded the ULN 21 times (6.8%), 41 times (13.2%), and 49 times (15.7%), respectively, indicating that LFTs generally remained below the ULN. Transient elevations were observed, which returned to normal limits within a week. Four episodes of rapid changes in LFT results were noted. Notably, between February 18 and 19, 2023 (during the 2nd infectious period), AST, ALT, and GGT levels showed marked elevations. During this period, UGI bleeding occurred, and LFTs results improved to near-normal levels when vancomycin administered on February 18, 2023, was discontinued. This finding suggests that the transient changes were attributable to vancomycin exposure and ischemic shock related to UGI bleeding.

Mild elevations were also observed on March 28, 2023, and December 18 and 29, 2024 following levofloxacin administration. These changes resolved spontaneously by the following day without any intervention, suggesting that herbal medications were unlikely to have contributed to these changes. From May 6, 2025, to May 20, 2025 (during the 10th infectious period), AST, ALT, and GGT levels increased following cefepime administration (May 2–9, 2025). LFT parameters stabilized when cefepime was discontinued and replaced with ceftazidime, indicating that these changes were due to cefepime rather than the herbal medications.

During vancomycin-associated hepatotoxicity, AST and ALT levels were approximately 5 times the ULN, and elevations may increase up to ten times the ULN.^[37,38]^ Moreover, values can increase > 20 times the ULN during ischemic shock^[39]^; in this patient, AST levels ranged from 140 to 1015 U/L and ALT levels from 230 to 1045 U/L. Levofloxacin-induced mild hepatotoxicity generally causes elevations of up to 3 times the ULN^[40,41]^; in this patient, AST levels ranged from 37 to 51 U/L and ALT levels from 44 to 80 U/L. Cefepime-induced hepatotoxicity typically increases AST and ALT levels reaching the mid-200s, and GGT levels between 300 to 700 U/L^[42]^; in this patient, AST levels ranged from 42 to 131 U/L, ALT levels from 36 to 115 U/L, and GGT levels from 15 to 25 U/L. In conclusion, these values fluctuated within the ranges reported in previous studies, supporting the finding that herbal medications was unlikely to have contributed to or accelerated antibiotic-induced liver injury.

During noninfectious periods, LFT parameters remained mostly below the ULN. Transient elevations in GGT levels were observed between the 1st and 2nd infectious periods (from January 16, 2023, to February 4, 2023) and between the 3rd and 4th infectious periods (from August 7, 2023, to November 4, 2023). These fluctuations resolved without any intervention, suggesting that they were unrelated to herbal medications or other drugs. After the 10th infectious period (from June 7, 2025), mild elevations in LFT parameters occurred following rosuvastatin administration for dyslipidemia. The values decreased after changing the statin type, indicating statin-induced fluctuations rather than the effects of herbal medications. Rosuvastatin-induced hepatotoxicity generally causes elevations in AST and ALT levels below 3 times the ULN, and increases of 5 to ten times the ULN have been reported when co-administered with other drugs.^[43,44]^ In this patient, AST ranged from 56 to 97 U/L and ALT from 48 to 81 U/L, which were within the expected ranges, supporting the conclusion that herbal medications did not exacerbate hepatic outcomes. The safety of long-term IPMC implementation for over 1 year in patients with end-stage renal disease and stroke has been reported in a previous study based on LFT results.^[45]^

#### 4.2.2. Renal function

Renal function was maintained within normal ranges throughout infectious and noninfectious periods. A transient BUN elevation was observed from February 18 to 19, 2023, following vancomycin administration. When vancomycin was discontinued on February 20, 2023, the BUN levels normalized, indicating vancomycin-related nephrotoxicity. Previous studies suggest that vancomycin-induced nephrotoxicity can increase BUN levels to approximately 65 mg/dL and SCr levels to 0.4 to 1.05 mg/dL and lower eGFR to 18 mL/minute/1.73 m^2^.^[46,47]^ In this patient, BUN levels ranged from 21 to 44 mg/dL. This change was within expected ranges, supporting the conclusion that herbal medications did not accelerate nephrotoxicity. The safety of long-term IPMC implementation for over 1 year in patients with end-stage renal disease and stroke has been reported in a previous study based on RFT results.^[45]^

### 4.3. Significance and limitations of the study

A major strength of this study is its longitudinal design, documenting the patient’s clinical course for more than 2 years. Unlike many case reports that are limited to short-term observations, this study provides a continuous record of infection recurrence, antibiotic prescriptions, and herbal medication use. Such a comprehensive documentation provides a rare opportunity to observe how treatment strategies evolve in response to changing inflammatory profiles.

Another strength is the detailed description of integrative management. By systematically recording the effects of conventional antibiotics and herbal medications, this case highlights the dynamic interplay between the 2 therapeutic options. The transition from broad-spectrum antibiotic dependence to herbal-dominated treatment is particularly noteworthy and illustrates the feasibility of reducing antibiotic exposure while maintaining inflammation control.

Furthermore, parallel monitoring of hematologic indices, such as CRP levels, NLR, and SIRI, adds significance. In this case, SIRI values remained persistently elevated (mean 19.1–32.5) during infectious periods, indicating patient’s chronic systemic immune-inflammatory activation. In contrast, NLR values consistently remained within the normal range, suggesting that preserved lymphocyte counts buffered against excessive acute inflammatory responses.^[48]^ Thus, SIRI reflected the systemic immune-inflammatory burden, whereas NLR captured the intensity of acute inflammatory responses. The ability to interpret these complementary markers in tandem represents a methodological strength of this study, underscoring the utility of multidimensional biomarker monitoring for long-term infection management.

Several clinically significant findings were also observed. First, the GCS and LOC showed recovery of consciousness, and meaningful improvements in motor function were observed in the MMT and FMA, suggesting that IPMC contributed to functional recovery. Considering that this patient had poor prognostic indicators with a high expected mortality rate, recovery of consciousness and motor function after 2 years of treatment underscores the clinical significance of IPMC. Second, because CRP levels below 2.0 mg/dL were managed with herbal medications alone, this threshold can be proposed as a criterion for herbal medication use during infectious periods. Third, management with herbal medication can reduce the resistance associated with long-term antibiotic exposure, disruption of the gut microbiota, nephrotoxicity, and risk of *Clostridioides difficile* infection. Finally, most LFTs and RFTs values remained within normal ranges, and transient elevations were due to antibiotics or other medications rather than herbal medications. This indicates that long-term co-administration of herbal medications with antibiotics, antiepileptics, or antihypertensive drugs is safe in patients with brainstem hemorrhage.

However, this study has some limitations. First, cognitive assessments using the K-MMSE and GDS could not be accurately performed because of the patient’s tracheostomy tube. Second, inconsistencies were noted in the neurological examinations. The intervals between the neurological assessments were not uniform. Furthermore, MMT scores recorded in the ward’s progress notes differed from those recorded by the Department of Rehabilitation Medicine. To address this issue, MMT scores recorded in the progress notes, which provided more detailed descriptions of the patient’s condition, were prioritized. Third, the patient’s inherently low baseline NLR could have masked an infection-related elevation, potentially maintaining the value within the normal range despite the underlying infection. Fourth, accurately assessing the degree of renal impairment using eGFR calculated from SCr levels was challenging. Due to severe muscle atrophy from quadriplegia, the patient’s SCr level was artificially low, leading to a significant overestimation of the eGFR.^[49]^ Fifth, herbal medications were not standardized but rather a formulation prepared in the hospital. To minimize this limitation, the hospital systematically prepared medicines using hGMP-certified herbal materials. Sixth, this retrospective study introduced potential inaccuracies in the results. FMA, GCS, and LOC scores at admission were estimated from the progress notes, which may have reduced the precision of the baseline assessments. Finally, as a single-patient case report, the findings cannot be generalized, and the lack of a control group makes it difficult to establish causality.

## 5. Conclusions

This case report describes a patient with brainstem hemorrhage who underwent IPMC for the management of posthemorrhagic functional impairment and complications, primarily recurrent infections. Regarding the efficacy of IPMC, improvements in neurological deficits and effective control of inflammation were observed. Notably, a potential hypothesis is that infection may be managed with herbal medications alone when CRP levels are below 2.0 mg/dL. Regarding the safety of IPMC, long-term administration of herbal medications did not result in hepatic or renal dysfunction.

Although this was a retrospective single-patient case report, the patient and caregiver expressed satisfaction with the treatment during hospitalization, and these findings provide a foundation for future prospective studies. Further research, including cohort studies and randomized controlled trials, is required to strengthen the evidence and validate the effectiveness of this approach.

## Acknowledgments

This research was supported by a grant from the Korea Health Technology R&D Project through the Korea Health Industry Development Institute (KHIDI), funded by the Ministry of Health & Welfare, Republic of Korea (RS-2024-00441603). This represents *direct monetary support* provided through the KHIDI by the Ministry of Health & Welfare, Republic of Korea.

## Author contributions

**Investigation:** Ye-Vin Lee, Jonghun Ahn, Euiju Lee, Chelin Park, Eui-Jin Son, Ja Yeon Jeong.

**Methodology:** Ye-Vin Lee, Jonghun Ahn, Euiju Lee, Chelin Park, Eui-Jin Son, Ja Yeon Jeong.

**Visualization:** Ye-Vin Lee, Jonghun Ahn, Eui-Jin Son.

**Writing – original draft:** Ye-Vin Lee, Jonghun Ahn, Chelin Park, Eui-Jin Son.

**Conceptualization:** Euiju Lee.

**Funding acquisition:** Euiju Lee.

**Project administration:** Euiju Lee.

**Writing – review & editing:** Euiju Lee.
